# Prostate-selective α antagonists increase fracture risk in prostate cancer patients with and without a history of androgen deprivation therapy: a nationwide population-based study

**DOI:** 10.18632/oncotarget.23828

**Published:** 2018-01-02

**Authors:** Wei-Heng Kao, Chang-Fu Kuo, I-Jun Chou, Lai-Chu See, Wen-Kuan Huang, Meng-Jiun Chiou, Weiya Zhang, Michael Doherty, Chun-Chieh Wang, Jun-Te Hsu, Hsien-Hsin Chen, Ji-Hong Hong

**Affiliations:** ^1^ Department of Radiation Oncology, Chang Gung Memorial Hospital, Taoyuan, Taiwan; ^2^ Division of Rheumatology, Allergy and Immunology, Department of Internal Medicine, Chang Gung Memorial Hospital, Taoyuan, Taiwan; ^3^ Division of Rheumatology, Orthopaedics and Dermatology, School of Medicine, University of Nottingham, Nottingham, UK; ^4^ Department of Paediatrics, Chang Gung Memorial Hospital, Taoyuan, Taiwan; ^5^ Department of Public Health, College of Medicine, Chang Gung University, Taoyuan, Taiwan; ^6^ Biostatistics Core Laboratory, Molecular Medicine Research Center, Chang Gung University, Taoyuan, Taiwan; ^7^ Division of Hematology-Oncology, Department of Internal Medicine, Chang Gung Memorial Hospital, Taoyuan, Taiwan; ^8^ Department of General Surgery, Chang Gung Memorial Hospital, Taoyuan, Taiwan; ^9^ Department of Medical Imaging and Radiological Science, Chang Gung University, Taoyuan, Taiwan; ^10^ Radiation Biology Research Center, Institute for Radiological Research, Chang Gung University/Chang Gung Memorial Hospital, Taoyuan, Taiwan

**Keywords:** prostate-selective α antagonists, prostate cancer, androgen deprivation therapy, fracture, population-based study

## Abstract

**Introductions:**

Prostate-selective α antagonists are recommended for relief of lower urinary tract symptoms in prostate cancer patients despite uncertainty of fracture risk as an addition to androgen deprivation therapy (ADT). The purpose of this study is to estimate fracture risk associated with these medications in prostate cancer patients who did and did not receive ADT.

**Methods:**

The Taiwan National Health Insurance database was used to identify prostate cancer patients. We identified all 90-day person-quarters exposed to and not exposed to prostate-selective α antagonists. A generalized estimating equation model was used to estimated adjusted odd ratios (ORs) and 95% confidence intervals (CIs) for fracture associated with prostate-selective α antagonists with consideration for confounding by indication bias using propensity score.

**Results:**

During 1997–2008, 16,601 persons received a diagnosis of prostate cancer, among whom 13,694 received ADT. Among prostate cancer patients receiving ADT, fracture was significantly more common in person-quarters with prostate-selective α antagonist use than in quarters without such treatment (OR, 1.08; 95% CI, 1.00–1.18). Prostate-selective α antagonist use was most strongly associated with femur fracture (OR, 1.22; 95% CI, 1.09–1.38), followed by skull fracture (OR, 1.29; 95% CIs: 0.93–1.80). Among patients who did not receive ADT, fracture was more common in person-quarters with prostate-selective α antagonist use than in those without medication use (OR, 1.19; 95% CI, 0.91–1.55).

**Conclusions:**

Prostate-selective α antagonist is associated with an increased fracture risk, particular for fractures in skull and femur. Patients should be well-informed on this potential risk before taking prostate-selective α antagonists.

## INTRODUCTION

Prostate cancer is the fifth most common male cancer in Taiwan [1]. Current guidelines recommend androgen deprivation therapy (ADT) as first-line neoadjuvant and adjuvant therapy in conjunction with radiotherapy for locally advanced prostate cancer and as the standard treatment for disseminated prostate cancer [2–4]. Despite these recommendations, the balance between the therapeutic benefits and adverse effects of ADT—such as insulin resistance, diabetes mellitus and increased risks of cardiovascular diseases, accelerated bone loss —has not been adequately studied [5–11].

Patients with prostate cancer frequently have urinary symptoms which can adversely affect quality of life. Such symptoms can be relieved by α antagonists. Prostate-selective α antagonists such as tamsulosin, silodosin (α_1A_ antagonists), and alfuzosin (α_1_ antagonists with uroselectivity) are believed to have a better safety profile than nonselective agents because they are less likely to result in side effects such as hypotension, syncope, and dizziness, which may predispose patients with prostate cancer—who are already at risk for osteoporosis because of androgen deprivation—to falls and fracture [12–16].

Results of studies on the safety of prostate-selective α antagonists for prostate cancer patients receiving androgen deprivation have been contradictory, particularly those related to the risks of falls and fracture [17–19]. In addition, there is limited evidence regarding fracture risk associated with prostate-selective α antagonists, with or without a history of ADT. Therefore, we estimated the effects of prostate-selective α antagonists on fracture risk among prostate cancer patients receiving ADT or not receiving ADT in Taiwan between 1997 and 2008.

## RESULTS

### Patient characteristics

**Figure 1:**
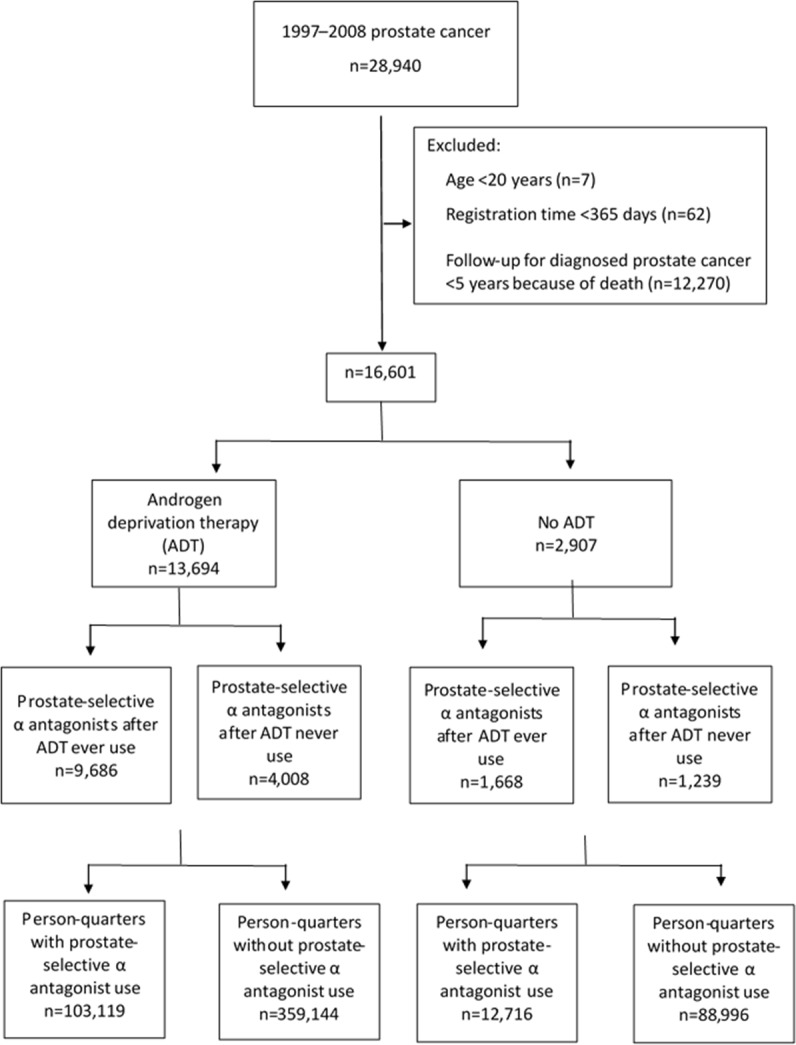
Flow of included patients for analyses with numbers of excluded observations

During 1997–2008, a total of 16,601 patients were eligible for this study. Among them, 13,694 of received ADT. Among patients receiving ADT, 9,686 (70.7%) used one or more types of prostate-selective α antagonist and 4,008 (29.3%) never used a prostate-selective α antagonist. Among patients without a history of ADT (n = 2907), 1668 had been prescribed prostate-selective α antagonists (Figure 1). The characteristics of the patients at diagnosis are shown in Table 1. The absolute standardized mean differences of the patients’ characteristics after propensity score weighting are listed in Supplementary Tables 9 and 10.

**Table 1:** Characteristics of study population

Characteristics	With androgen deprivation therapy					Without androgen deprivation therapy				
	Any prostate-selective α antagonist use (n=9,686)		No prostate-selective α antagonist use (n=4,008)		p value	Any prostate-selective α antagonist use (n=1,668)		No prostate-selective α antagonist use (n=1,239)		p value
Age (years) (mean ± standard deviation)	73.02 ± 7.27		70.73 ± 8.17		<0.0001	70.52 ± 7.69		67.62 ± 9.10		<0.0001
Charlson Comorbidity Index†	3.85 ± 2.14		3.57 ± 2.15		<0.0001	3.50 ± 1.65		3.11 ± 1.59		<0.0001
≤3	5,561	(57.41)	2,668	(66.57)	<0.0001	1037	(62.17)	916	(54.92)	<0.0001
>3	4,125	(42.59)	1,340	(33.43)		631	(37.83)	323	(19.36)	
Comorbidities										
Hypertension^†^	5,318	(54.90)	1,893	(47.23)	<0.0001	909	(54.50)	556	(33.33)	<0.0001
Osteoporosis^‡^	911	(9.41)	271	(6.76)	<0.0001	170	(10.19)	79	(4.74)	0.0003
Medication use, No. (%)^†^										
Calcium channel blockers	5,229	(53.99)	1,853	(46.23)	<0.0001	866	(51.92)	511	(30.64)	<0.0001
ACE inhibitors or ARB	3,799	(39.22)	1,343	(33.51)	<0.0001	625	(37.47)	383	(22.96)	0.0002
β blockers	3,687	(38.07)	1,343	(33.51)	<0.0001	679	(40.71)	387	(23.20)	<0.0001
α blockers^§^	7,373	(76.12)	2,855	(71.23)	<0.0001	1189	(71.28)	763	(45.74)	<0.0001
Hydrazinophthalazine	784	(8.09)	327	(8.16)	0.8999	126	(7.55)	61	(3.66)	0.0043
K+ sparing diuretics	1,002	(10.34)	313	(7.81)	<0.0001	129	(7.73)	79	(4.74)	0.1602
Loop diuretics	3,129	(32.30)	1,196	(29.84)	0.0048	522	(31.29)	371	(22.24)	0.4347
Thiazide diuretics	2,596	(26.80)	893	(22.28)	<0.0001	423	(25.36)	228	(13.67)	<0.0001
Benzodiazepines	7,304	(75.41)	2,705	(67.49)	<0.0001	1252	(75.06)	762	(45.68)	<0.0001
Bisphosphonates	83	(0.86)	16	(0.40)	0.0040	7	(0.42)	4	(0.24)	0.6741
Glucocorticoids	5316	(54.88)	1983	(49.48)	<0.0001	905	(54.26)	579	(34.71)	<0.0001
Narcotics	2,655	(27.41)	1,082	(27.00)	0.6201	495	(29.68)	341	(20.44)	0.2045
Overactive-bladder medications	3,025	(31.23)	1,081	(26.97)	<0.0001	516	(30.94)	306	(18.35)	0.0002
Proton pump inhibitors	1,386	(14.31)	458	(11.43)	<0.0001	268	(16.07)	160	(9.59)	0.0176
Statins	1,312	(13.55)	495	(12.35)	0.0601	280	(16.79)	163	(9.77)	0.0071
5-α-reductase inhibitors	2,603	(26.87)	895	(22.33)	<0.0001	117	(7.01)	60	(3.60)	0.0155
NSAIDs	8,995	(92.87)	3,561	(88.85)	<0.0001	1550	(92.93)	1,076	(64.51)	<0.0001
Insulin	502	(5.18)	183	(4.57)	0.1319	80	(4.80)	58	(3.48)	0.8854
Anticoagulants	693	(7.15)	245	(6.11)	0.0281	150	(8.99)	117	(7.01)	0.6776
Anticonvulsants	1,307	(13.49)	332	(8.28)	<0.0001	237	(14.21)	112	(6.71)	<0.0001
Lipid lowering agents	1,790	(18.48)	686	(17.12)	0.0591	353	(21.16)	217	(13.01)	0.0143
Treatment^¶^										
Radiotherapy	4,730	(48.83)	1,310	(32.68)	<0.0001	526	(31.53)	190	(11.39)	<0.0001
Radical prostatectomy	1,070	(11.05)	1,244	(31.04)	<0.0001	694	(41.61)	720	(43.17)	<0.0001
Place of residence, No. (%)					<0.0001					0.3899
Urban	2,843	(29.35)	1,366	(34.08)		547	(32.79)	428	(25.66)	
Suburban	2,640	(27.26)	1,030	(25.70)		471	(28.24)	329	(19.72)	
Rural	4,002	(41.32)	1519	(37.90)		616	(36.93)	448	(26.86)	
Unknown	201	(2.08)	93	(2.32)		34	(2.04)	34	(2.04)	
Income level, No. (%)					<0.0001					<0.0001
Quintile 1	2460	(25.40)	896	(22.36)		467	(28.00)	285	(23.00)	
Quintile 2	1376	(14.21)	523	(13.05)		240	(14.39)	168	(13.56)	
Quintile 3	2251	(23.24)	874	(21.81)		318	(19.06)	228	(18.40)	
Quintile 4	1797	(18.55)	731	(18.24)		364	(21.82)	242	(19.53)	
Quintile 5	1765	(18.22)	965	(24.08)		278	(16.67)	302	(24.37)	
Unknown	37	(0.38)	19	(0.47)		1	(0.06)	14	(1.13)	
Occupation, No. (%)					<0.0001					<0.0001
Dependent of insured individual	2,479	(25.59)	1,023	(25.52)		401	(24.04)	271	(16.25)	
Civil servant, teacher, military personnel, and veteran	1,085	(11.20)	435	(10.85)		212	(12.71)	149	(8.93)	
Non-manual workers and professionals	746	(7.70)	497	(12.40)		163	(9.77)	198	(11.87)	
Manual workers	3,144	(32.46)	1,186	(29.59)		446	(26.74)	327	(19.60)	
Other	2,232	(23.04)	867	(21.63)		446	(26.74)	294	(17.63)	

^†^ Diagnosed during the 3 years before prostate cancer diagnosis.

^‡^ Diagnosed any time before prostate cancer diagnosis.

^§^ Tamsulosin, silodosin, alfuzosin were excluded.

^¶^ At any time.

### Fracture risk in patients with prostate cancer and in patients with ADT

Standardized fracture risk was higher among prostate cancer patients than among persons without cancer, with a SIR (95% CI) of 1.39 (1.27-1.52) (Supplementary Table 4). Among prostate cancer patients, fracture risk of patients with a history of ADT was higher than those without ADT with a HR (95% CI) of 1.41 (1.26-1.57) (Supplementary Table 5).

### Fracture risk associated with prostate-selective α antagonist use

Overall there were 563,975 person-quarters for all prostate cancer patients. In patients with a history of ADT, fracture events occurred in 4,012 person-quarters (0.87%) of 462,263 person-quarters. Fractures were significantly more frequent in person-quarters with prostate-selective α antagonist use (1,125/103,119 person-quarters) than in those without such use (2,887/359,144 person-quarters) among prostate cancer patients with a history of ADT. Prostate-selective α antagonist use was associated with a crude OR for fracture of 1.28 (95% CI, 1.19-1.39) and an adjusted OR of 1.09 (95% CI, 1.00-1.18). The three most common fracture sites were the femur (n=1,796); tibia, fibula, and foot (n=950); and radius and ulna (n=644). Among fracture sites, prostate-selective α antagonist use was most strongly associated with femur fracture (crude OR, 1.41; 95% CI, 1.27–1.57; adjusted OR, 1.22; 95% CI, 1.09–1.38), followed by skull fracture with borderline significance (Table 2).

**Table 2:** Fracture risk in prostate cancer patients with a history of androgen deprivation therapy with and without prostate-selective α antagonist use based on fracture diagnosed in emergency and inpatient departments using propensity score weighting method

Fracture sites	Full sample (n=462,263)	Person-quarters with prostate-selective α antagonist use (n=103,119)		Person-quarters without prostate-selective α antagonist use (n=359,144)		Crude OR^a^ (95% CI)		Adjusted OR^b^ (95% CI)	
All fractures	4,012	1,125	(1·09)	2,887	(0·80)	1.28	(1.19-1.39)^*^	1.08	(1.00-1.18)^*^
Skull	194	60	(0·06)	134	(0·04)	1.49	(1.10-2.03)^*^	1.29	(0.93-1.80)
Vertebrae	585	179	(0·17)	406	(0·11)	1.41	(1.16-1.71)^*^	1.09	(0.88-1.35)
Rib	330	88	(0·09)	242	(0·07)	1.22	(0.94-1.57)	1.00	(0.76-1.32)
Pelvis	64	17	(0·02)	47	(0·01)	1.14	(0.64-2.01)	0.98	(0.54-1.78)
Clavicle	161	30	(0·03)	131	(0·04)	0.82	(0.55-1.22)	0.66	(0.44-1.01)
Scapula	35	7	(0·01)	28	(0·01)	0.89	(0.39-2.06)	0.77	(0.32-1.84)
Humerus	634	150	(0·15)	484	(0·13)	1.04	(0.86-1.26)	0.88	(0.72-1.08)
Radius and ulna	644	148	(0·14)	496	(0·14)	1.00	(0.82-1.22)	0.82	(0.67-1.01)
Hand	340	89	(0·09)	251	(0·07)	1.23	(0.96-1.58)	1.16	(0.89-1.52)
Femur	1,796	548	(0·53)	1248	(0·35)	1.41	(1.27-1.57)^*^	1.22	(1.09-1.38)^*^
Tibia, fibula, and foot	950	254	(0·25)	696	(0·20)	1.27	(1.09-1.47)^*^	1.12	(0.95-1.31)

^a^ Adjusted for age.

^b^ Using inverse probability of treatment weights of propensity scores (medical utilization, Charlson Comorbidity Index, hypertension, osteoporosis, calcium channel blocker, ACE inhibitor, ARB, diuretics-K+ sparing, diuretics-loop diuretics, diuretics-thiazide, β blockers, α blockers (tamsulosin, silodosin, alfuzosin were excluded), benzodiazepine, bisphosphonates, glucocorticoids, narcotics, overactive bladder medication, proton pump inhibitors, statin, 5-α-reductase inhibitors, hydrazinophthalazine, NSAID, insulin, anticoagulants, anticonvulsants, lipid lowering agents, echocardiography (EKG), bone mineral density test, bone scan, cardiac stress test, CT head, radiotherapy, radical prostectomy, place of residence, income levels, occupation).

^*^p<0.05.

In the analysis of prostate cancer patients without a history of ADT, 552 person-quarters with fracture were identified among 101,712 person-quarters. There were more fractures in person-quarters with prostate-selective α antagonist use (105/12,716 person-quarters) than in quarters without prostate-selective α antagonist use (447/88,996 person-quarters) (crude OR, 1.43; 95% CI, 1.13–1.80; adjusted OR, 1.19; 95% CI, 0.91–1.55). The most frequent fracture sites were the femur (n=225); tibia, fibula, and foot (n=181); and radius and ulna (n=89), and only tibia, fibula, and foot fracture risk significantly increased with prostate-selective α antagonist use (crude OR, 1.63; 95% CI, 1.09–2.44; adjusted OR, 1.54; 95% CI, 1.00–2.36). Prostate-selective α antagonist use in patients without a history of ADT was borderline associated with femur fracture (crude OR, 1.51; 95% CI, 1.07–2.11; adjusted OR, 1.42; 95% CI, 0.98–2.06) (Table 3).

**Table 3:** Fracture risk in prostate cancer patients without a history of androgen deprivation therapy with and without prostate-selective α antagonist use based on fracture diagnosed in emergency and inpatient departments using propensity score weighting method

Fracture sites	Full sample (n=101,712)	Person-quarters with prostate-selective α antagonist use (n=12,716)		Person-quarters without prostate-selective α antagonist use (n=88,996)		Crude OR^a^ (95% CI)		Adjusted OR^b^ (95% CI)	
Overall fracture	552	105	(0·83)	447	(0·50)	1·43	(1·13-1·80)^*^	1.19	(0.91-1.55)
Skull	32	8	(0·06)	24	(0·03)	2·02	(0·91-4·49)	1.36	(0.45-4.13)
Vertebrae	67	11	(0·09)	56	(0·06)	1·06	(0·52-2·13)	0.76	(0.35-1.63)
Rib	54	9	(0·07)	45	(0·05)	1·36	(0·67-2·73)	1.13	(0.50-2.55)
Pelvis	12	2	(0·02)	10	(0·01)	1·45	(0·29-7·26)	1.10	(0.21-5.88)
Clavicle	25	4	(0·03)	21	(0·02)	1·32	(0·45-3·93)	0.95	(0.29-3.09)
Scapula	5	0	(0·00)	5	(0·01)		N/A		N/A
Humerus	68	11	(0·09)	57	(0·06)	1·19	(0·62-2·27)	0.82	(0.39-1.72)
Radius and ulna	89	10	(0·08)	79	(0·09)	0·82	(0·42-1·59)	0.75	(0.36-1.54)
Hand	57	11	(0·09)	46	(0·05)	1·56	(0·76-3·18)	1.9	(0.88-4.10)
Femur	225	46	(0·36)	179	(0·20)	1·51	(1·07-2·11)^*^	1.42	(0.98-2.06)
Tibia, fibula, and foot	181	37	(0·29)	144	(0·16)	1·63	(1·09-2·44)^*^	1.54	(1.00-2.36)^*^

^a^ Adjusted for age.

^b^ Using inverse probability of treatment weights of propensity scores (medical utilization, Charlson Comorbidity Index, hypertension, osteoporosis, calcium channel blocker, ACE inhibitor, ARB, diuretics-K+ sparing, diuretics-loop diuretics, diuretics-thiazide, β blockers, α blockers (tamsulosin, silodosin, alfuzosin were excluded), benzodiazepine, bisphosphonates, glucocorticoids, narcotics, overactive bladder medication, proton pump inhibitors, statin, 5-α-reductase inhibitors, hydrazinophthalazine, NSAID, insulin, anticoagulants, anticonvulsants, lipid lowering agents, echocardiography (EKG), bone mineral density test, bone scan, cardiac stress test, CT head, radiotherapy, radical prostectomy, place of residence, income levels, occupation).

^*^p<0.05.

### Additional analyses

We expanded the case definition of fracture to outpatient, inpatient and emergency procedures for fracture and repeated the analysis. As shown in Supplementary Tables 6 and 7, prostate-selective α antagonist use was associated with an adjusted OR of 1.05 (95% CI, 0.97–1.13) for any fracture among patients with ADT use. In patients without a history of ADT, prostate-selective α antagonist use was associated with an adjusted OR of 1.10 (95% CI, 0.86–1.41). For femur fracture, OR was 1.2 (95% CI, 1.07-1.35) among patients with ADT use. For patients without ADT use, the adjusted OR (95% CI) for femur fracture was 1.50 (1.05-2.13). In Supplementary Table 8, for patients with ADT use history who are older than or equal to 73 years old, adjusted OR of any fracture is 1.09 (95% CI, 1.00-1.20) and OR is 1.00 (95% CI, 0.80-1.25) for those younger than 73 years old. Adjusted OR are 0.8 (95%CI 0.42-1.54, age < 73 year-old) and 1.26 (95% CI 0.96-1.69, ≥73 year-old) for patients without ADT history.

## DISCUSSION

### Principal findings

The Prostate Cancer Survivorship Care Guidelines of the American Society of Clinical Oncology recommend that physicians consider α-blockers for slow stream urinary dysfunction [20]. However, the safety of prostate-selective α antagonists remains a matter of debate. In this study, use of prostate-selective α antagonists was associated with a 1.08-fold increase in fracture risk among prostate cancer patients with a history of ADT. Analysis of prostate cancer patients without a history of ADT use revealed a similar increase in fracture risk (OR, 1.19). In addition, prostate-selective α antagonists were associated with increased risks of skull (OR, 1.29) and femur fracture (OR, 1.22), which suggests that postural hypotension associated with these drugs was the main predisposing factor. Our findings indicate that fracture risk is higher among prostate-selective α antagonist users than among nonusers, irrespective of ADT use, presumably because of the adverse effects of postural hypotension.

A previous study reported a 2.8-fold increase in the risk of fracture requiring hospitalization among prostate cancer patients receiving ADT [10]. Among patients who received GnRH agonists, fracture risk was 38% higher than for patients who did not receive GnRH agonists and 103% higher than for patients who underwent orchiectomy [11]. In the present study, fracture incidence was higher among patients who received ADT than among those who did not (adjusted HR, 1.41) in Supplementary Table 5. In fracture site analysis, only the risk of femoral fracture was significantly increased, regardless of ADT exposure. The risk of skull fracture was borderline-significantly higher only in patients with a history of ADT. However, there was small difference in the magnitude of relative risks associated with prostate-selective α antagonists in patients who received ADT (OR: 1.08) and those who did not (OR: 1.19). This might be due to higher fracture percentage in patients who received ADT without prostate-selective α antagonists use (ADT vs. no ADT= 0.8% vs. 0.5%). Although the difference in relative risk was modest, the absolute increase in events in personal-quarters for patients who received ADT was much higher than for those who did not receive ADT, because patients receiving ADT had more risk factors for fracture at baseline [10, 11]. This could result in more fracture-related deaths among these patients [10].

### Comparison with previous findings

Two large population-based studies reported that use of nonselective α antagonists was associated with increased risk of hypotension-related adverse events and hip/femur fracture within 4 months after the start of therapy [12]. These adverse effects were more common during the initial stage after starting a new treatment (for both prostate-selective and nonselective antagonists) [21]. However fracture risk was not associated with use of modified-release doxazosin in The Health Improvement Network (THIN) UK database case-control study, [22] and a Danish nationwide case-control study reported no association between fracture risk and use of α antagonists (for both prostate-selective and nonselective antagonists) [23]. A recent population-based retrospective cohort study reported a temporal association between tamsulosin use and severe hypotension during the first 8 weeks after initiating treatment and 8 weeks after restarting treatment, [17] and a matched cohort study found significantly higher risks of falling and fracture in patients treated with alfuzosin, tamsulosin, or silodosin [18]. However, change in blood pressure on standing did not significantly differ between tamsulosin and placebo in a relatively small double-blind phase III study, [24] and rates of dizziness, headache, and hypotension were relatively low in a large cohort questionnaire study [25].

### Study strengths and limitations

This nationwide population-based study included newly diagnosed prostate cancer patients followed-up for over a decade. With propensity score weighting in each person-quarter, we considered complex prescription decision-making for prostate-selective α antagonists, which is based on changes in patient condition. In addition, we conducted multiple sensitivity analyses which all indicate an increased risk for femur fracture in prostate cancer patients using prostate-selective α antagonist with or without ADT. Therefore, although the increase in fracture risk associated with prostate-selective α antagonist was modest, it is nevertheless a genuine risk, particularly for femur fracture that should be considered by physicians.

This study has several limitations. Firstly, it is possible that some fractures were related to disease progression in patients with bony metastases. However, in our study, we only included the patients with minimum survival time of 5 years to reduce the impact of the pathologic fracture and based on the Denmark cohort study, the 5-year-survival probability of the prostate cancer patients with whenever bony metastases was 2.7% (95% CI 2.2-3.4) [26]. Secondly, our data of radiotherapy course had no details of prostate irradiation only or prostate plus pelvic irradiation. Nevertheless the cumulative incidence of insufficiency fracture in pelvic irradiation was relative low with 6.8% [27]. Although we didn't adjust the irradiation specific to pelvic region, we still adjusted the factor of receiving radiotherapy or not for the fracture result. Third, we didn't have daily blood pressure with direct connection with our theory of drug-induced hypotension and fracture. In another way, we try to use these patients’ hypertensive medications to eliminate the impact. Fourth, some potential confounding factors may not have been measured in our study, such as cancer stage, tumor grade, relatively rare comorbidities and limited mobility [13]. However, these factors are unlikely to change the increase in fracture risk associated with prostate-selective α antagonist use in prostate cancer patients [18].

## MATERIALS AND METHODS

### Design and setting

This study was approved by the institutional review board of Chang Gung Memorial Hospital in Taiwan (approval number 104-7041B). The primary data source was the Taiwan National Health Insurance (NHI) database. The NHI system was established in 1995 and is a single-payer insurance. Approximately 23 million beneficiaries were registered in 2013 with a coverage rate of 99.5% [28]. The accuracy of the recorded diagnoses is high, and the validity of the database has been confirmed in multiple studies [29–33]. The database is anonymized before its release for research use.

In Taiwan, patients with major diseases, including solid and haematological malignancies, receive a waiver of medical co-payment (catastrophic illness certificate). The unique personal identifier, diagnosis, demographic data, application date, and other data for patients receiving a medical waiver are recorded in the Registry of Patients with Catastrophic Illnesses, which we used to identify patients with prostate cancer in the present study (International Classification of Disease, ninth revision, clinical modification [ICD-9-CM] code, 185.0).

To ascertain the validity of prostate cancer diagnoses in the NHI database, we linked the NHI with the National Cancer Registry, which served as the reference standard. We identified 40,303 incident cases of prostate cancer between 2001 and 2012, as indicated by a catastrophic illness certificate for prostate cancer. Agreement between the NHI database and National Cancer Registry was good, with a sensitivity of 0.92, a specificity of 1, a positive predictive value of 0.93, and a negative predictive value of 1 [34].

### Patient population and covariates

**Figure 2:**
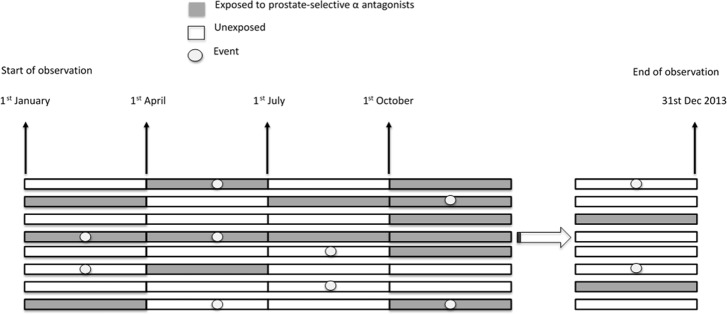
Schematic presentation of person-quarter design

We established a cohort of patients aged 20 years or older who received a new diagnosis of prostate cancer between 1 January 1997 and 31 December 2008 (Figure 1). A minimum of one year of observation before the date of diagnosis and five years of follow-up were required for inclusion in the analysis [26]. Patients were grouped according to use of ADT (including gonadotropin-releasing hormone agonists (GnRH), anti-androgen agents, and bilateral orchiectomy). Each calendar year was partitioned into four quarters for each patient and each year after ADT (Figure 2). The analytic unit was thus one quarter. We identified all person-quarters with exposures to tamsulosin, alfuzosin, or silodosin and those without such exposures.

The patients’ demographics, comorbidities, medication use, treatment of prostate cancer, and health care use were analysed as covariates [18]. Patient demographics included age, gender, place of residence, income level, and occupation. The Charlson comorbidity index, [35] was used to evaluate patient comorbidity burden in relation to 17 diagnostic conditions three years before the cancer diagnosis. Other comorbidities, including hypertension three years before cancer diagnosis and osteoporosis at any time before diagnosis, were also evaluated. Twenty-two types of medications were assessed [18].

### Study outcomes

Our primary outcome was any or site-specific fracture. The unit of analysis was a person-quarter. We used ICD-9-CM codes to identify fractures recorded in emergency and inpatient departments and procedure codes for fracture management in inpatient, and emergency departments and confirmed with ICD-9-CM codes. Fractures were classified according to sites. Fractures were evaluated in each person-quarter and therefore one patient can have multiple events. In addition, we performed a sensitivity analysis using the aforementioned procedure codes for fracture management not only in emergency and inpatient departments, but also the codes in outpatient clinics, and then classified fracture location. The full code lists for ADT, outcome definition and covariates are shown in the Supplementary Tables 1, 2 and 3.

### Statistical analysis

In our primary analyses, we used multivariable logistic regression to model the relation between fracture and use of prostate-selective α antagonists among patients with prostate cancer, with or without ADT. The prescription of prostate-selective α antagonist may subject to the “confounding by indication” as a result of non-random treatment allocation [36] which was accounted for by utilising propensity score weighting methods [37]. The propensity score is the predicted probability that a patient is prescribed prostate-selective α antagonist during a quarter using logistic regression to model this group as a function of potential confounders of the grouping status and study outcomes [37, 38]. For each person-quarter, an inverse probability of treatment weights of specific propensity score was calculated using logistic regression with covariates pertinent to the first date of the quarter, as determined by analysis of the previous quarter and balance covariates (medical utilization, Charlson Comorbidity Index, hypertension, osteoporosis, calcium channel blocker, ACE inhibitor, ARB, diuretics-K+ sparing, diuretics-loop diuretics, diuretics-thiazide, beta blocker, alpha blocker, benzodiazepine, bisphosphonates, glucocorticoids, narcotics, overactive bladder medication, proton pump inhibitors, statin, 5-α-reductase inhibitors, hydrazinophthalazine, NSAID, insulin, anticoagulants, anticonvulsants, lipid lowering agents, echocardiography (EKG), bone mineral density test, bone scan, cardiac stress test, CT head, radiotherapy, radical prostatectomy, place of residence, income levels, occupation) across the two study groups. To account for intra-individual correlation in outcomes across quarters, we used multivariable logistic regression with a generalized estimating equation model to calculate crude odd ratios (ORs), 95% CIs, and adjusted estimates. The correlation structure was based on autoregressive model.

We further performed additional analysis. Firstly, we compared the overall and site-specific fracture incidence between patients with prostate cancer with the general population. Standardized incidence ratio (SIR) for fracture was calculated. The SIR is the ratio of the actual fracture number of patients with cancer in this study to the expected fracture number of patients with cancer based on age- and time-specific incidence rates in 5-year age intervals in the general population (Supplementary Table 4). To compare the risk of fracture between people with or without ADT, we further used Cox proportional hazards model to estimate hazard ratio (HR) for fracture, adjusting for the aforementioned covariates. ADT use was time dependent variable (Supplementary Table 5). Secondly, we conducted a sensitivity analysis using an alternative case definition for fractures, which not only included fracture events identified from emergency or inpatient records but also outpatient records (Supplementary Tables 6 and 7). Otherwise we stratified our study population by median age (73 years old) to examine the interaction between age and prostate-selective α antagonist (Supplementary Table 8). The results of additional analysis were shown in the supplements. The entire analysis was done using SAS V 9.4.

## CONCLUSION

Use of prostate-selective α antagonists is associated with a 29% increased risk of skull fracture and 22% increased risk for femur fracture. Physicians should balance the benefits of relieving lower urinary tract symptoms with the potential risk of hypotension, falls, and fracture in this population at high risk of osteoporosis. Patients receiving prostate-selective α antagonists should be fully informed of fracture risk and preventive measures should be undertaken in order to reduce this risk.

## SUPPLEMENTARY MATERIALS TABLES








