# Supplementary material for: Prostate-selective α antagonists increase fracture risk in prostate cancer patients with and without a history of androgen deprivation therapy: a nationwide population-based study
Source: Oncotarget. 2018 Jan 2;9(4):5263–73. doi: 10.18632/oncotarget.23828 (PMC5797048; doi:10.18632/oncotarget.23828)
Supplement: Supplementary file 1 [file oncotarget-09-5263-s001.pdf]

Prostate-selective α antagonists increase fracture risk in prostate cancer patients with and without a history of androgen deprivation therapy: a nationwide population-based study

SUPPLMENTARY METARIALS

Supplementary Table 1: Drugs used for androgen deprivation therapy

Gonadotropin-releasing hormone analogues	Leuprolide acetate, Goserelin acetate, Triptorelin acetate, Degarelix, Abiraterone acetate
Anti-androgens	Flutamide, Bicalutamide, Cyproterone acetate

Supplementary Table 2: Twenty-two types of medications adjusted for in propensity score analysis of probability of prostate-selective α antagonist prescription

See Supplementary File 1

Supplementary Table 3: Procedure codes and ICD-9-CM codes for fracture sites

Fracture location	Procedure codes	ICD-9-CM
Skull	61020C, 64008C, 64101B, 64102B	800-804
Vertebrae	64042C, 64160B, 64248C, 43034C, 43036C	805-806
Rib	67003B, 64017C	807
Pelvis	64042C, 64161B, 64236B, 61010C, 61011C, 61012C, 64248C, 95010B	808
Clavicle	61017C, 64015C, 64016C, 52011C	810
Scapula	61017C, 64235B, 43037C, 43038C, 95009B	811
Humerus	64043C, 64239B, 61013C, 61016C, 61017C, 95002C, 95003C, 95009B, 95014C, 95015C	812
Radius and ulna	61014C, 61015C, 61016C, 64032B, 64044C, 64271C, 52012C, 95002C, 95003C, 95014C, 95015C	813
Hand	64267C, 64035C, 64036C, 64045C, 64047C, 64049C, 43016C, 43017C, 52015C, 64027C, 95001C, 95013C	814-817
Femur	64028C, 64034B, 64041C, 61007C, 61009C, 61012C, 64029B, 64030B, 95004C, 95005C, 95010B, 43012C, 43013C, 95016C, 95017C	820-821
Tibia, fibula, and foot	61001C, 61002C, 61003C, 61004C, 61005C, 61006C, 61008C, 64031C, 64034B, 64043C, 64046C, 64048C, 64049C, 64239B, 64272C, 64273C, 92206B, 43010C, 43011C, 52015C, 64027C, 64035C, 64036C, 95004C, 95005C, 95006C, 95007C, 95008C, 95013C, 95016C, 95017C	822-827

Supplementary Table 4: Standardised incidence ratio for fracture in prostate cancer patients versus the general population

Fracture sites	Observed	Expected	Standarised incidence ratio	
Overall fracture	479	346	1·39	(1·27-1·52)*
Skull	14	13	1·07	(0·63-1·81)
Vertebrae	89	65	1·36	(1·10-1·67)*
Rib	27	29	0·92	(0·63-1·34)
Pelvis	4	4	1·07	(0·40-2·85)
Clavicle	14	10	1·37	(0·81-2·31)
Scapula	1	1	0·72	(0·10-5·11)
Humerus	13	11	1·18	(0·69-2·03)
Radius and ulna	29	17	1·70	(1·18-2·45)*
Hand	13	13	1·00	(0·58-1·72)
Femur	72	53	1·37	(1·09-1·73)*
Tibia, fibula, and foot	36	32	1·11	(0·80-1·54)

Supplementary Table 5: Risk of fracture in prostate cancer patients with and without a history of androgen deprivation therapy

Fracture sites	HR[a] (95% CI)		HR[b] (95% CI)	
Overall fracture	1·49	(1·34-1·66)*	1·41	(1·26-1·57)*
Skull	1·28	(0·87-1·87)	1·18	(0·79-1·75)
Vertebrae	1·62	(1·23-2·14)*	1·55	(1·17-2·06)*
Rib	1·33	(0·98-1·78)	1·25	(0·92-1·69)
Pelvis	1·27	(0·65-2·47)	1·22	(0·62-2·42)
Clavicle	1·47	(0·95-2·26)	1·35	(0·86-2·11)
Scapula	1·59	(0·62-4·10)	1·75	(0·66-4·65)
Humerus	1·93	(1·49-2·51)*	1·74	(1·34-2·27)*
Radius and ulna	1·54	(1·22-1·94)*	1·45	(1·14-1·84)*
Hand	1·37	(1·02-1·84)*	1·36	(1·00-1·84)*
Femur	1·53	(1·31-1·79)*	1·44	(1·23-1·68)*
Tibia, fibula and foot	1·19	(1·00-1·42)*	1·12	(0·93-1·34)

[a] Adjusted for age.
[b] Adjusted for age, Charlson Comorbidity Index, chronic lung disease, dementia, diabetes, hypertension, osteoporosis, calcium channel blockers, ACE inhibitors, ARB, K+ sparing diuretics, loop diuretics, thiazide diuretics, β blockers, α blockers (tamsulosin, silodosin, alfuzosin were excluded), benzodiazepines, bisphosphonates, glucocorticoids, narcotics, medications for overactive bladder, proton pump inhibitors, statins, 5-α-reductase inhibitors, hydrazinophthalazine, NSAID, insulin, anticoagulants, anticonvulsants, lipid lowering agents.

Supplementary Table 6: Fractures of prostate cancer patients with androgen deprivation therapy with/without prostate-selective α antagonist use in emergency, inpatient and outpatient departments using propensity score weighting method

Person-quarters	Full sample (n=462,263)	Person-quarters with prostate-selective α antagonist use (n=103,119)		Person-quarters without prostate-selective α antagonist use (n=359,144)		Crude OR[a] (95% CI)		Adjusted OR[b] (95% CI)	
Overall fracture	4,817	1,307	(1.27)	3,510	(0.98)	1·24	(1·15-1·33)*	1.05	(0.97-1.13)
Skull	196	62	(0.06)	134	(0.04)	1·55	(1·14-2·12)*	1.34	(0.96-1.88)
Vertebrae	593	181	(0.18)	412	(0.11)	1·40	(1·16-1·70)*	1.09	(0.88-1.35)
Rib	347	90	(0.09)	257	(0.07)	1·18	(0·92-1·51)	0.98	(0.75-1.29)
Pelvis	64	17	(0.02)	47	(0.01)	1·14	(0·64-2·01)	0.98	(0.54-1.78)
Clavicle	186	36	(0.03)	150	(0.04)	0·85	(0·59-1·22)	0.72	(0.49-1.05)
Scapula	40	8	(0.01)	32	(0.01)	0·88	(0·40-1·92)	0.77	(0.34-1.74)
Humerus	917	213	(0.21)	704	(0.20)	1·02	(0·86-1·20)	0.86	(0.72-1.02)
Radius and ulna	941	212	(0.21)	729	(0.20)	0·97	(0·82-1·15)	0.81	(0.68-0.96)
Hand	622	148	(0.14)	474	(0.13)	1·09	(0·90-1·33)	0.97	(0.79-1.20)
Femur	1,996	596	(0.58)	1,399	(0.39)	1·38	(1·24-1·53)*	1.2	(1.07-1.35)*
Tibia, fibula and foot	1,356	353	(0.34)	1,003	(0.28)	1·23	(1·08-1·41)*	1.09	(0.95-1.26)

[a] Adjusted for age.
[b] Using inverse probability of treatment weights of propensity scores (medical utilization, Charlson Comorbidity Index, hypertension, osteoporosis, calcium channel blocker, ACE inhibitor, ARB, diuretics-K+ sparing, diuretics-loop diuretics, diuretics-thiazide, β blockers, α blockers (tamsulosin, silodosin, alfuzosin were excluded), benzodiazepine, bisphosphonates, glucocorticoids, narcotics, overactive bladder medication, proton pump inhibitors, statin, 5-α-reductase inhibitors, hydrazinophthalazine, NSAID, insulin, anticoagulants, anticonvulsants, lipid lowering agents, echocardiography (EKG), bone mineral density test, bone scan, cardiac stress test, CT head, radiotherapy, radical prostectomy, place of residence, income levels, occupation).
*$p<0.05$.

Supplementary Table 7: Fractures of prostate cancer patients without androgen deprivation therapy with/without prostate-selective α antagonist use in emergency, inpatient and outpatient departments using propensity score weighting method

Person-quarters	Full sample (n=101,712)	Person-quarters with prostate-selective α antagonist use (n=12,716)		Person-quarters without prostate-selective α antagonist use (n=88,996)		Crude OR[a] (95% CI)		Adjusted OR[b] (95% CI)	
Overall fracture	680	122	(0.96)	558	(0.63)	1·37	(1·10-1·70)*	1·10	(0·86-1·41)
Skull	32	8	(0.06)	24	(0.03)	2·01	(0·90-4·46)	1·36	(0·45-4·15)
Vertebrae	69	12	(0.09)	57	(0.06)	1·18	(0·60-2·32)	0·80	(0·37-1·70)
Rib	57	9	(0.07)	48	(0.05)	1·26	(0·63-2·54)	1·06	(0·48-2·34)
Pelvis	12	2	(0.02)	10	(0.01)	1·75	(0·37-8·24)	1·10	(0·20-5·98)
Clavicle	27	4	(0.03)	23	(0.03)	1·26	(0·44-3·67)	0·88	(0·27-2·83)
Scapula	6	0	(0.00)	6	(0.01)	N/A		N/A	
Humerus	112	13	(0.10)	99	(0.11)	0·81	(0·45-1·46)	0·62	(0·33-1·18)
Radius and ulna	129	13	(0.10)	116	(0.13)	0·71	(0·39-1·27)	0·64	(0·34-1·20)
Hand	108	19	(0.15)	89	(0.10)	1·45	(0·86-2·45)	1·12	(0·63-2·00)
Femur	253	53	(0.42)	200	(0.22)	1·58	(1·14-2·19)*	1·50	(1·05-2·13)*
Tibia, fibula and foot	252	48	(0.38)	204	(0.23)	1·54	(1·08-2·19)*	1·33	(0·91-1·95)

[a] Adjusted for age.
[b] Using inverse probability of treatment weights of propensity scores (medical utilization, Charlson Comorbidity Index, hypertension, osteoporosis, calcium channel blockers, ACE inhibitors, ARB, diuretics-K+ sparing, diuretics-loop diuretics, diuretics-thiazide, β blockers, α blockers (tamsulosin, silodosin, alfuzosin were excluded), benzodiazepine, bisphosphonates, glucocorticoids, narcotics, overactive bladder medications, proton pump inhibitors, statins, 5-α-reductase inhibitors, hydrazinophthalazine, NSAID, insulin, anticoagulants, anticonvulsants, lipid lowering agents, echocardiography (EKG), bone mineral density test, bone scan, cardiac stress test, CT head, radiotherapy, radical prostectomy, place of residence, income levels, occupation).
*$p<0.05$.

Supplementary Table 8: Fracture risk in prostate cancer patients with/ without a history of androgen deprivation therapy with and without prostate-selective α antagonist use based on fracture diagnosed in emergency and inpatient departments using propensity score weighting method stratified by age

All fractures		Full sample	Person-quarters with prostate-selective α antagonist use		Person-quarters without prostate-selective α antagonist use		Crude OR[a] (95% CI)		Adjusted OR[b] (95% CI)	
With ADT	Age <73	676/ 119,721	135/ 19,672	(0.69)	541/ 100,049	(0.45)	1.25	(1.02-1.54)*	1.00	(0.80-1.25)
	Age ≥73	3,336/ 342,542	990/ 83477	(1.19)	2,346/ 259,095	(0.91)	1.29	(1.19-1.40)*	1.09	(1.00-1.20)*
Without ADT	Age <73	123/ 39,954	11/ 3,455	(0.32)	112/ 36,499	(0.31)	0.99	(0.54-1.81)	0.8	(0.42-1.54)
	Age ≥73	429/ 61,758	94/ 9,261	(1.02)	335/ 52,497	(0.64)	1.53	(1.19-1.98)	1.26	(0.94-1.69)

[a] Adjusted for age.
[b] Using inverse probability of treatment weights of propensity scores (medical utilization, Charlson Comorbidity Index, hypertension, osteoporosis, calcium channel blocker, ACE inhibitor, ARB, diuretics-K+ sparing, diuretics-loop diuretics, diuretics-thiazide, β blockers, α blockers (tamsulosin, silodosin, alfuzosin were excluded), benzodiazepine, bisphosphonates, glucocorticoids, narcotics, overactive bladder medication, proton pump inhibitors, statin, 5-α-reductase inhibitors, hydrazinophthalazine, NSAID, insulin, anticoagulants, anticonvulsants, lipid lowering agents, echocardiography (EKG), bone mineral density test, bone scan, cardiac stress test, CT head, radiotherapy, radical prostectomy, place of residence, income levels, occupation).
*$p<0.05$.

Supplementary Table 9: Characteristics of study population with androgen deprivation therapy use

See Supplementary File 2

Supplementary Table 10: Characteristics of study population without androgen deprivation therapy use

See Supplementary File 3
