# Supplementary material for: Prostate-selective α antagonists increase fracture risk in prostate cancer patients with and without a history of androgen deprivation therapy: a nationwide population-based study
Source: Oncotarget. 2018 Jan 2;9(4):5263–73. doi: 10.18632/oncotarget.23828 (PMC5797048; doi:10.18632/oncotarget.23828)
Supplement: Supplementary file 2 [file oncotarget-09-5263-s002.docx]

**Supplementary Table 2: Twenty-two types of medications adjusted for in propensity score analysis of probability of prostate-selective α antagonist prescription**

| **Prostate-selective α antagonists** | **ALFUZOSIN, SILODOSIN, TAMSULOSIN,** |
| --- | --- |
| ACEI | CAPTOPRIL, LISINOPRIL, ENALAPRIL, IMIDAPRIL, FOSINOPRIL, BENAZEPRIL, RAMIPRIL, PERINDOPRIL, QUINAPRIL, CILAZAPRIL |
| Anticoagulants | APIXABAN, DABIGATRAN, DALTEPARIN SODIUM, ENOXAPARIN SODIUM, FONDAPARINUX SODIUM, HEPARIN, NADROPARINE CALCIUM, PHENINDIONE, RIVAROXABAN, TINZAPARIN SODIUM, WARFARIN |
| Anticonvulsants | VIGABATRIN, PHENOBARBITAL, LEVETIRACETAM, PHENYTOIN, CARBAMAZEPINE, CLONAZEPAM, LAMOTRIGINE, VALPROATE SODIUM, TOPIRAMATE,  GABAPENTIN, VALPROIC ACID, OXCARBAZEPINE,  DIVALPROEX, TIAGABINE HYDROCHLORIDE, ZONISAMIDE,  PREGABALIN, PRIMACLONE  DIPROPYLACETAMIDE  PERAMPANEL |
| ARB | LOSARTAN, OLMESARTAN, VALSARTAN, IRBESARTAN, TELMISARTAN, CANDESARTAN, EPROSARTAN, AZILSARTAN |
| α blockers (tamsulosin, silodosin, alfuzosin were excluded) | PRAZOSIN, DOXAZOSIN, TERAZOSIN, PHENOXYBENZAMINE, PHENTOLAMINE |
| β blockers | PROPRANOLOL, OXPRENOLOL, ATENOLOL, TIMOLOL, PINDOLOL, METOPROLOL, NADOLOL, ACEBUTOLOL, BISOPROLOL, CARTEOLOL, BETAXOLOL, METIPRANOLOL, ALPRENOLOL, BUPRANOLOL, ESMOLOL |
| Bisphosphonate | ETIDRONATE, CLODRONATE, ALENDRONATE, PAMIDRONATE, RISEDRONATE, ZOLEDRONATE, IBANDRONATE |
| BZD | ALPRAZOLAM, BROMAZEPAM, CHLORDIAZEPOXIDE, CLONAZEPAM, CLOXAZOLAM, DIAZEPAM, FLUDIAZEPAM, ESTAZOLAM, FLUNITRAZEPAM, FLURAZEPAM, LORAZEPAM, LORMETAZEPAM, MEDAZEPAM, MIDAZOLAM, NIMETAZEPAM, NITRAZEPAM, NORDAZEPAM, OXAZEPAM, PRAZEPAM, TEMAZEPAM, TRIAZOLAM, ZOLPIDEM, ZOPICLONE, ESZOPICLONE, ZALEPLON |
| Calcium channel blockers | NIFEDIPINE, NICARDIPINE, NITRENDIPINE, AMLODIPINE, ISRADIPINE, FELODIPINE, LACIDIPINE, NIMODIPINE, LERCANIDIPINE, BENIDIPINE, BARNIDIPINE, VERAPAMIL, DILTIAZEM, |
| Drugs for reactive bladder | FLAVOXATE, OXYBUTYNIN, SOLIFENACIN, TOLTERODINE, TROSPIUM, |
| Glucocorticoids | HYDROCORTISONE, CORTISONE, PREDNISOLONE, METHYLPREDNISOLONE, TRIAMCINOLONE, BETAMETHASONE, DEXAMETHASONE, |
| Hydrazinophthalazine | HYDRALAZINE |
| Insulins | REGULAR INSULIN, INSULIN ASPART, INSULIN DETEMIR, INSULIN GLARGINE, INSULIN GLULISINE, INSULIN HUMAN, INSULIN LISPRO, NPH INSULIN |
| K+ sparing diuretics | AMILORIDE, TRIAMTERENE, SPIRONOLACTONE, EPLERENONE |
| Lipid lowering agents | ACIPIMOX, AMLODIPINE, ATORVASTATIN, BEZAFIBRATE, CHOLESTYRAMINE, CLOFIBRATE, COLESTIPOL HCL, DEXTRAN SULFATE SODIUM, ETOFIBRATE, EZETIMIBE, FENOFIBRATE, FLUVASTATIN SODIUM, GEMFIBROZIL, LOVASTATIN, NIACIN, NICERITROL, NICOFURANOSE, NICOMOL, PITAVASTATIN, PROBUCOL, ROSUVASTATIN CALCIUM, SIMFIBRATE, SIMVASTATIN, SOYSTEROL |
| Loop diuretics | FUROSEMIDE, BUMETANIDE, ETHACRYNIC |
| Narcotics | APOMORPHINE, MORPHINE, HYDROMORPHONE, TRAMADOL, FENTANYL, OXYCODONE, MEPERIDINE, CODEINE |
| Nonsteroidal anti-inflammatory drugs | ACECLOFENAC, ACEMETACIN, ALCLOFENAC, ALMINOPROFEN, BENZYDAMINE HCL, CELECOXIB, DICLOFENAC, ETODOLAC, ETORICOXIB, FENBUFEN, FENOPROFEN (CALCIUM), FLUFENAMATE ALUMINUM, FLUFENAMIC ACID, FLURBIPROFEN, IBUPROFEN, INDOMETHACIN, KETOPROFEN, KETOROLAC, TROMETHAMINE, MECLOFENAMATE, MEFENAMIC ACID, MELOXICAM, MEPIRIZOLE, NABUMETONE, NAPROXEN, NIFLUMIC ACID, NIMESULIDE, PHENYLBUTAZONE, PIROXICAM, ROFECOXIB, SULINDAC, TENOXICAM, TIAPROFENIC ACID, TIARAMIDE, TOLFENAMIC ACID, TOLMETIN |
| Proton pump inhibitors | OMEPRAZOLE, LANSOPRAZOLE, PANTOPRAZOLE, RABEPRAZOLE, ESOMEPRAZOLE, DEXLANSOPRAZOLE |
| Statins | LOVASTATIN, PRAVASTATIN, SIMVASTATIN, ATORVASTATIN, ROSUVASTATIN, PITAVASTATIN, FLUVASTATIN, |
| Thiazide diuretics | METOLAZONE, HYDROCHLOROTHIAZIDE, TRICHLORMETHIAZIDE, BENZYLHYDROCHLOROTHIAZIDE, BUTHIAZIDE, BENDROFLUMETHIAZIDE |
| 5-α-reductase inhibitors | FINASTERIDE, DUTASTERIDE, |
