# Supplementary material for: Prostate-selective α antagonists increase fracture risk in prostate cancer patients with and without a history of androgen deprivation therapy: a nationwide population-based study
Source: Oncotarget. 2018 Jan 2;9(4):5263–73. doi: 10.18632/oncotarget.23828 (PMC5797048; doi:10.18632/oncotarget.23828)
Supplement: Supplementary file 3 [file oncotarget-09-5263-s003.docx]

**Supplementary Table 9: Characteristics of study population with androgen deprivation therapy use**

|  | **Propensity Score Weighting** | | | | | | | | |  |
| --- | --- | --- | --- | --- | --- | --- | --- | --- | --- | --- |
|  | **Before** | | |  | | **After** | | | |  |
| **Characteristics** | **Person-quarters with prostate-selective α antagonist use (n=103,119)** | **Person-quarters without prostate-selective α antagonist use (n=359,144)** | **ASMD** | |  | | **Person-quarters with prostate-selective α antagonist use (n=103,119)** | **Person-quarters without prostate-selective α antagonist use (n=359,144)** | **ASMD** |  |
| Age (years) (mean ± standard deviation) | 78.57 ± 7.37 | 76.76 ± 7.98 | 0.2355 | |  | | 78.57 ± 7.37 | 78.53 ± 4.02 | 0.0074 |  |
| Charlson Comorbidity Index † | 2.69 ± 1.83 | 2.16 ± 1.83 | 0.2881 | |  | | 2.69 ± 1.83 | 2.70 ± 1.01 | -0.0095 |  |
| ≤3 | 79.04 | 85.13 | -0.1592 | |  | | 79.04 | 77.98 | 0.0260 | |
| >3 | 20.96 | 14.87 |  | |  | | 20.96 | 22.02 |  | |
| Comorbidities |  |  |  | |  | |  |  |  | |
| Hypertension† | 39.02 | 34.57 | 0.0924 | |  | | 39.02 | 39.41 | -0.0079 | |
| Osteoporosis‡ | 20.42 | 15.83 | 0.1195 | |  | | 20.42 | 20.52 | -0.0024 | |
| Medication use, No· (%)† |  |  |  | |  | |  |  |  | |
| Calcium channel blockers | 35.67 | 32.04 | 0.0769 | |  | | 35.67 | 36.08 | -0.0085 | |
| ACE inhibitors or ARB | 30.44 | 25.81 | 0.1030 | |  | | 30.44 | 30.91 | -0.0102 | |
| β blockers | 18.24 | 16.62 | 0.0431 | |  | | 18.24 | 18.39 | -0.0036 | |
| α blockers | 15.88 | 23.87 | -0.2013 | |  | | 15.88 | 16.25 | -0.0101 | |
| Hydrazinophthalazine | 1.41 | 1.34 | 0.0062 | |  | | 1.41 | 1.42 | -0.0006 | |
| K+ sparing diuretics | 4.10 | 3.42 | 0.0361 | |  | | 4.10 | 4.25 | -0.0071 | |
| Loop diuretics | 12.75 | 9.15 | 0.1155 | |  | | 12.75 | 13.13 | -0.0113 | |
| Thiazide diuretics | 11.88 | 10.04 | 0.0588 | |  | | 11.88 | 12.00 | -0.0038 | |
| Benzodiazepines | 41.24 | 32.44 | 0.1833 | |  | | 41.24 | 41.96 | -0.0145 | |
| Bisphosphonates | 3.18 | 1.93 | 0.0790 | |  | | 3.18 | 3.28 | -0.0060 | |
| Glucocorticoids | 16.40 | 13.43 | 0.0835 | |  | | 16.40 | 16.73 | -0.0087 | |
| Narcotics | 8.89 | 5.96 | 0.1118 | |  | | 8.89 | 9.23 | -0.0121 | |
| Overactive-bladder medications | 4.77 | 4.44 | 0.0156 | |  | | 4.77 | 4.84 | -0.0034 | |
| Proton pump inhibitors | 5.92 | 4.43 | 0.0672 | |  | | 5.92 | 6.09 | -0.0078 | |
| Statins | 9.66 | 9.04 | 0.0211 | |  | | 9.66 | 9.82 | -0.0056 | |
| 5-α-reductase inhibitors | 1.01 | 0.45 | 0.0664 | |  | | 1.01 | 1.03 | -0.0023 | |
| NSAIDs | 43.32 | 36.93 | 0.1306 | |  | | 43.32 | 44.01 | -0.0140 | |
| Insulin | 3.79 | 3.27 | 0.0286 | |  | | 3.79 | 3.95 | -0.0082 | |
| Anticoagulants | 3.31 | 2.47 | 0.0696 | |  | | 3.31 | 3.46 | -0.0107 | |
| Anticonvulsants | 7.12 | 5.43 | 0.0504 | |  | | 7.12 | 7.40 | -0.0082 | |
| Lipid lowering agents | 11.47 | 10.84 | 0.0200 | |  | | 11.47 | 11.65 | -0.0057 | |
| Treatment¶ |  |  |  | |  | |  |  |  | |
| Radiotherapy | 40.42 | 33.62 | 0.1412 | |  | | 40.42 | 40.48 | -0.0012 | |
| Radical prostatectomy | 4.22 | 19.81 | -0.4940 | |  | | 4.22 | 4.21 | -0.0001 | |
| Place of residence, No· (%) |  |  | 0.0686 | |  | |  |  | 0.0286 | |
| Urban | 28.69 | 31.63 |  | |  | | 28.69 | 28.81 |  | |
| Suburban | 27.46 | 27.26 |  | |  | | 27.46 | 27.61 |  | |
| Rural | 41.98 | 39.27 |  | |  | | 41.98 | 42.08 |  | |
| Unknown | 1.87 | 1.84 |  | |  | | 1.87 | 1.50 |  | |
| Income level, No· (%) |  |  | 0.2213 | |  | |  |  | 0.0859 | |
| Quintile 1 | 10.58 | 16.10 |  | |  | | 10.58 | 10.59 |  | |
| Quintile 2 | 32.41 | 28.16 |  | |  | | 32.41 | 32.57 |  | |
| Quintile 3 | 10.10 | 12.61 |  | |  | | 10.10 | 10.30 |  | |
| Quintile 4 | 30.62 | 24.99 |  | |  | | 30.62 | 30.71 |  | |
| Quintile 5 | 15.92 | 17.55 |  | |  | | 15.92 | 15.83 |  | |
| Unknown | 0.37 | 0.59 |  | |  | | 0.37 | 0 |  | |
| Occupation, No· (%) |  |  | 0.1825 | |  | |  |  | 0.0115 | |
| Dependent of insured individual | 29.42 | 28.36 |  | |  | | 29.42 | 29.50 |  | |
| Civil servant, teacher, military personnel, and veteran | 21.84 | 18.53 |  | |  | | 21.84 | 22.04 |  | |
| Non-manual workers and professionals | 3.51 | 5.72 |  | |  | | 3.51 | 3.50 |  | |
| Manual workers | 29.66 | 26.83 |  | |  | | 29.66 | 29.79 |  | |
| Other | 15.57 | 20.56 |  | |  | | 15.57 | 15.17 |  | |

ASMD = absolute standardized mean difference
