# Supplementary material for: Prostate-selective α antagonists increase fracture risk in prostate cancer patients with and without a history of androgen deprivation therapy: a nationwide population-based study
Source: Oncotarget. 2018 Jan 2;9(4):5263–73. doi: 10.18632/oncotarget.23828 (PMC5797048; doi:10.18632/oncotarget.23828)
Supplement: Supplementary file 4 [file oncotarget-09-5263-s004.docx]

**Supplementary Table 10: Characteristics of study population without androgen deprivation therapy use**

|  | **Propensity Score Weighting** | | | | | | | | |  |
| --- | --- | --- | --- | --- | --- | --- | --- | --- | --- | --- |
|  | **Before** | | |  | **After** | | | |  |  |
| **Characteristics** | **Person-quarters with prostate-selective α antagonist use (n=12,716)** | **Person-quarters without prostate-selective α antagonist use (n=88,996)** | **ASMD** |  | **Person-quarters with prostate-selective α antagonist use (n=12,716)** | **Person-quarters without prostate-selective α antagonist use (n=88,996)** | **ASMD** |  |  |  |
| Age (years) (mean ± standard deviation) | 76.80 ± 8.14 | 73.84 ± 8.93 | 0.3464 |  | 76.80 ± 8.14 | 76.67 ± 3.16 | 0.0203 |  |  |  |
| Charlson Comorbidity Index † | 2.11 ± 1.59 | 1.52 ± 1.50 | 0.3779 |  | 2.11 ± 1.59 | 2.10 ± 0.60 | 0.0043 |  |  |  |
| ≤3 | 84.70 | 91.80 | -0.2217 |  | 84.70 | 85.32 | -0.0172 | | | |
| >3 | 15.30 | 8.20 |  |  | 15.30 | 14.68 |  | | | |
| Comorbidities |  |  |  |  |  |  |  | | | |
| Hypertension† | 39.97 | 34.93 | 0.1043 |  | 39.97 | 41.21 | -0.0252 | | | |
| Osteoporosis‡ | 18.90 | 13.00 | 0.1615 |  | 18.90 | 18.96 | -0.0016 | | | |
| Medication use, No· (%)† |  |  |  |  |  |  |  | | | |
| Calcium channel blockers | 38.50 | 32.69 | 0.1216 |  | 38.50 | 39.03 | -0.008 | | | |
| ACE inhibitors or ARB | 30.83 | 27.37 | 0.0761 |  | 30.83 | 31.81 | -0.0212 | | | |
| β blockers | 19.83 | 16.79 | 0.0787 |  | 19.83 | 19.76 | 0.0017 | | | |
| α blockers | 16.75 | 13.38 | 0.0945 |  | 16.75 | 17.84 | -0.0287 | | | |
| Hydrazinophthalazine | 1.18 | 1.19 | -0.0013 |  | 1.18 | 1.24 | -0.0059 | | | |
| K+ sparing diuretics | 3.92 | 2.82 | 0.0608 |  | 3.92 | 4.07 | -0.0079 | | | |
| Loop diuretics | 11.06 | 6.42 | 0.1651 |  | 11.06 | 11.34 | -0.0087 | | | |
| Thiazide diuretics | 13.13 | 10.07 | 0.0955 |  | 13.13 | 13.64 | -0.0152 | | | |
| Benzodiazepines | 44.83 | 29.62 | 0.3184 |  | 44.83 | 45.34 | -0.0104 | | | |
| Bisphosphonates | 0.17 | 0.14 | 0.0070 |  | 0.17 | 0.23 | -0.0135 | | | |
| Glucocorticoids | 15.74 | 11.44 | 0.1257 |  | 15.74 | 16.25 | -0.0140 | | | |
| Narcotics | 7.05 | 4.71 | 0.0992 |  | 7.05 | 7.31 | -0.0102 | | | |
| Overactive-bladder medications | 4.36 | 3.68 | 0.0349 |  | 4.36 | 4.62 | -0.0125 | | | |
| Proton pump inhibitors | 6.54 | 4.63 | 0.0829 |  | 6.54 | 6.57 | -0.0014 | | | |
| Statins | 13.21 | 11.26 | 0.0594 |  | 13.21 | 12.91 | 0.0090 | | | |
| 5-α-reductase inhibitors | 2.46 | 0.42 | 0.1718 |  | 2.46 | 2.36 | 0.0069 | | | |
| NSAIDs | 45.06 | 36.27 | 0.1797 |  | 45.06 | 45.94 | -0.0177 | | | |
| Insulin | 3.16 | 2.19 | 0.0601 |  | 3.16 | 3.35 | -0.0107 | | | |
| Anticoagulants | 3.59 | 2.67 | 0.0526 |  | 3.59 | 3.68 | -0.0049 | | | |
| Anticonvulsants | 8.44 | 4.81 | 0.1463 |  | 8.44 | 8.50 | -0.0024 | | | |
| Lipid lowering agents | 15.18 | 13.13 | 0.0588 |  | 15.18 | 14.96 | 0.0062 | | | |
| Treatment¶ |  |  |  |  |  |  |  | | | |
| Radiotherapy | 35.97 | 17.91 | 0.4159 |  | 35.97 | 36.04 | -0.0014 | | | |
| Radical prostatectomy | 17.29 | 51.32 | -0.7677 |  | 17.29 | 17.57 | -0.0073 | | | |
| Place of residence, No· (%) |  |  | 0.0353 |  |  |  | 0.0186 | | | |
| Urban | 31.72 | 32.99 |  |  | 31.72 | 31.12 |  | | | |
| Suburban | 29.19 | 27.85 |  |  | 29.19 | 29.2 |  | | | |
| Rural | 37.50 | 37.43 |  |  | 37.50 | 38.16 |  | | | |
| Unknown | 1.59 | 1.73 |  |  | 1.59 | 1.45 |  | | | |
| Income level, No· (%) |  |  | 0.2131 |  |  |  | 0.0545 | | | |
| Quintile 1 | 9.86 | 13.43 |  |  | 9.86 | 10.09 |  | | | |
| Quintile 2 | 39.06 | 30.98 |  |  | 39.06 | 38.52 |  | | | |
| Quintile 3 | 19.84 | 19.46 |  |  | 19.84 | 20.52 |  | | | |
| Quintile 4 | 17.58 | 18.69 |  |  | 17.58 | 17.85 |  | | | |
| Quintile 5 | 13.53 | 16.78 |  |  | 13.53 | 13.01 |  | | | |
| Unknown | 0.12 | 0.66 |  |  | 0.12 | 0 |  | | | |
| Occupation, No· (%) |  |  | 0.2197 |  |  |  | 0.0248 | | | |
| Dependent of insured individual | 26.90 | 26.84 |  |  | 26.90 | 27.16 |  | | | |
| Civil servant, teacher, military personnel, and veteran | 28.95 | 21.14 |  |  | 28.95 | 28.64 |  | | | |
| Non-manual workers and professionals | 5.65 | 8.41 |  |  | 5.65 | 5.19 |  | | | |
| Manual workers | 23.28 | 23.71 |  |  | 23.28 | 23.91 |  | | | |
| Other | 15.22 | 19.89 |  |  | 15.22 | 15.10 |  | | | |

ASMD = absolute standardized mean difference
